# Prof-in-a-Box: using internet-videoconferencing to assist students in the gross anatomy laboratory

**DOI:** 10.1186/1472-6920-6-55

**Published:** 2006-11-15

**Authors:** Stephen J Moorman

**Affiliations:** 1 Department of Neuroscience and Cell Biology, Robert Wood Johnson Medical School, 675 Hoes Lane West, Piscataway, NJ 08854 USA

## Abstract

**Background:**

The optimal learning environment for gross anatomy is the dissection laboratory. The Prof-in-a-Box (PiB) system has been developed where an anatomist using distance-learning technologies 'helps' students in a dissection laboratory at a different site.

**Methods:**

The PiB system consists of: (1) an anatomist in his/her office with a computer and video camera; (2) a computer and 2 video cameras in the lab; (3) iChat AV software; (4) a secure server to host the PiB-student 'consultation'. The PiB system allows the students and faculty to interact via audio and video providing an environment where questions can be asked and answered and anatomical structures can be identified 'at a distance' in real-time. The PiB system was set up at a prosected cadaver and made available for student use during 'office hours'.

**Results:**

25–30% of the students used the PiB system. Anatomical structures were identified, questions answered and demonstrations given 'at a distance' using the system. Students completed an optional questionnaire about the PiB system at the end of the semester. Results of the questionnaire indicate that the students were enthusiastic about the PiB system and wanted its use to be expanded in the future.

**Conclusion:**

Many of the functions of a faculty member in the gross anatomy dissection laboratory can be performed 'at a distance' using the PiB system. This suggests that a geographically dispersed faculty could assist in providing instruction in the dissection labs at multiple medical schools without needing to be physically present.

## Background

We have a problem in gross anatomy: Within the next decade there will be a critical national shortage of PhD-level faculty trained to teach gross anatomy [1]-the way we teach will have to change. Effective use of educational theory and distance-learning technologies to provide lectures (delivered by geographically dispersed faculty) to students at numerous schools is one way to continue to provide medical students with instruction in gross anatomy when this shortage becomes reality. However, the optimal learning environment for gross anatomy is the dissection laboratory [2-6] where students learn to recognize anatomical structures and their relationships *in situ*. To date, no one has demonstrated that it is possible to use distance-learning technologies to provide instruction in a dissection laboratory setting. Historically, this laboratory dissection experience has been supervised and guided by trained faculty in a setting with a significantly lower faculty-student ratio (1:20 at the home school) than that for the lectures (1:170 at the same school). In the dissection lab, the faculty serve as a resource to (1) answer questions, (2) help students identify structures, and (3) perform aspects of the dissections for the students when warranted. When the faculty shortage materializes, the quality of the laboratory experience will suffer as the faculty-student ratio in the dissection laboratory shifts toward that of the lecture.

This problem might be avoided by the development of a system where a trained anatomist using distance-learning technologies 'helps' students in a dissection laboratory at a different site. At least two of the three functions (1 & 2) of a faculty member in the dissection laboratory might be performed 'at a distance' using currently available distance-learning technologies. If this system is successful, the number of faculty needed "on-site" would be reduced to the number needed to perform the third function in the dissection lab. This could result in a geographically dispersed faculty able to provide both the lecture and laboratory experience for the students at numerous medical schools.

A **P**rof-**i**n-a-**B**ox system (PiB) has been developed that consists of three components: (1) a secure server to 'host' a videoconference; (2) a trained anatomist in his/her office with a computer and video camera; (3) a computer with two video cameras adjacent to a cadaver in the dissection lab. This system used iChat-AV software to allow the anatomist to see the students and the dissection via the cameras in the lab. The camera in the faculty office allowed the students to see the faculty member, providing a more personal aspect to the interaction. The software allowed the students and faculty to interact via live audio and video providing an environment where questions could be asked and answered and anatomical structures could be identified 'at a distance'.

## Methods

Prior to choosing computers for the PiB system, the following requirements were established (see Discussion):

Secure (encrypted) transmission

Built-in, cross-platform videoconference capability

No additional software to buy.

Cross-platform control of the computer in the laboratory from the office.

Sufficient camera resolution to identify anatomical structures

### Server

Since none of the commercial (free) instant messaging servers (MSN^®^, AOL^®^, and Yahoo^®^) that support videoconferences are secure, an in-house, secure server was set up to host the PiB videoconferences. The iChat server is a Jabber^® ^server that supports SSL Certificates and encryption and is an integral part of the Apple OSX v10.4 server software. The server consisted of the following hardware/software combination:

Dual Processor G5 computer (Apple Computers)

Dual 2.7 GHz PowerPC G5 processors, 2 GB SDRAM, 380 GB Hard Drive

OS-X 10.4 Server Software

Unlimited client license

iChat/Jabber^® ^server enabled

21" LCD Display (Apple Computers)

100 Mb Ethernet connection

The Information Services and Technology (IST) office at the university granted permission to connect this computer to the local network and have it function as a server provided it did not distribute IP addresses. IST also agreed to provide a 'static' IP address and domain name for the server. In order to facilitate future collaborations, IST also enabled data throughput on the university firewall for the ports specified in the iChat server documentation.

### Office Equipment

To avoid the need to find a Jabber^® ^client for a Windows operating system that supports secure videoconferences, an iMac computer was used. The most recent version of the Apple OS (v10.4) supports secure videoconferences through the iChat software included with the computer. Therefore, the following in the office:

20" iMac (Apple Computers)

1.8 GHz PowerPC G5 processor, 1 GB SDRAM, WIFI 802.11b/g card, 150 GB Hard Drive

iSight FireWire video camera (Apple Computers)

FireWire cable

OS-X v10.4

iChat-AV software (included as part of OS-X v10.4)

Apple Remote Desktop software v2.2

100 Mb Ethernet connection

The iSight camera and Apple Remote Desktop software were not included in the standard purchase of an iMac computer. The iSight camera is 'plug-and-play' and fully compatible with iChat. The Apple Remote Desktop (ARD) software was purchased to control the computer in the laboratory using the computer in the office (see discussion).

### Dissection Laboratory Equipment

Prior to setting up a system in the lab, a standard video camera (e.g., the iSight) was evaluated to determine if it had sufficient resolution to identify anatomical structures remotely. In a preliminary test using just bones, the resolution using the iSight camera was good enough to identify small structures such as tubercles. However, the working distance for the camera was 4–6 inches to give the necessary magnification. An inexpensive miniDV camera with FireWire output had higher resolution and a longer working distance than the iSight camera making the miniDV camera a better choice. The boom arm of a fluorescent drafting light was modified to hold the miniDV camera. This allowed the camera to be mounted either directly to the dissecting table or to the laboratory bench top adjacent to the dissecting table.

In the gross anatomy course, one cadaver is dissected by a faculty member. This prosected cadaver is available for the students to inspect and study outside of scheduled class hours. The following computer and peripheral equipment were set up at the prosected cadaver in the gross anatomy dissection lab.

Mac-Mini (Apple Computers)

1.42 GHz PowerPC G4 processor, 512 MB SDRAM, Bluetooth card, WIFI 802.11b/g card, 80 GB Hard Drive

17" monitor (Dell Computers)

iSight FireWire video camera (Apple Computers)

FireWire Cable

Optura-30 miniDV camera

FireWire Cable

Drafting light boom arm

FireWire (IEEE 1394) 3-port hub

USB Speakers

OS-X v10.4

iChat AV software (included as part of OS-X v10.4)

100 Mb Ethernet connection

This system, nicknamed Dr. PiB, sat on the laboratory bench at one end of the dissection table. The iSight camera, mounted on the monitor, was fixed in place and allowed the students at the prosected cadaver to be seen. The boom arm for the miniDV camera was mounted at the 'head' of the dissection table using the standard drafting light screw-mount. From this location, the students could position the miniDV camera on the boom arm over the cranial half of the prosected cadaver. By moving the boom arm to the 'foot' of the table, the camera could be positioned over the caudal half of the cadaver. Using the ARD software, remotely switching between the two cameras was possible. The Mac-Mini has only one FireWire port and only a very small internal speaker. Using the FireWire hub enabled both cameras to be connected to the computer simultaneously. The USB speakers allowed the student to hear the faculty member without leaving the dissection table.

### General Methods

In preparation for the project, the prosected cadaver was not inspected by the faculty member prior to any PiB session. In that way, the only information the faculty member had about the identity of a structure was limited to what could be 'seen' through the camera.

The same faculty member (the author) was available for 'consultation' via the PiB system one afternoon (1:30–4:30) a week on an afternoon when there were no other scheduled classes. Dr. PiB turned itself on and logged in to the iChat server automatically. The iChat software on Dr. PiB was configured to automatically accept invitations for videoconferences by running the 'Terminal' application, typing "defaults write com.apple.ichat AutoAcceptVCInvitations 1" and restarting the computer. A videoconference was initiated with Dr. PiB from the office and the conference was kept active for the entire 3-hour period. The iChat window on Dr. PiB was minimized when there was no one actively engaged in a consultation. When a student indicated that s/he had a question (usually by saying "Dr. PiB?"), the iChat window was made visible using the ARD software. Whenever a new student came to the cadaver, s/he was reminded that the faculty member was available through Dr. PiB to answer any questions s/he had.

The number of students who used the prosected cadaver was tracked, as were the times they arrived at the cadaver and the times they left. The questions they asked were logged, as were and the structures identified or assisted in identifying using the PiB system. In addition, any system problems that were encountered were logged. At the end of the gross anatomy course, all students in the course were provided with the opportunity to answer a brief, on-line, optional questionnaire about the PiB system.

## Results

The PiB system was deployed in the laboratory for 12 weeks of the 17-week semester that gross anatomy was in session. During this time, 60 students (out of 160 enrolled in the course) used the PiB system to 'consult' with the professor in the box. The identity of 30 structures was confirmed, 26 structures were identified and 14 questions were answered for the students using the PiB system. In addition, one demonstration of the movements of the digits of the hand was done using the PiB system.

The structures identified included the following:

Gemelli muscles

Obturator internus muscle

Dorsal Root Ganglion

Dorsal ramus

Ventral ramus

Common interosseous artery

Obturator nerve

Adductor muscles

Saphenous nerve

Coronary arteries

Coronary sinus

Lymph node on hilum of lung

Internal thoracic artery and vein

Umbilical ligaments

Left and middle colic arteries

Subcostal nerve

Ilioinguinal nerve

Branches of the internal iliac artery

Branches of external carotid artery

Medial pterygoid muscle

63 students completed the optional evaluation. The follow is a summary of the responses to the questions.

Question #1 – How often did you use the PiB?

a) Hardly at all 22

b) A couple of times 13

c) Often 1

d) Frequently 2

e) Almost every week 0

f) Not applicable 22

Question #2 – Was the PiB useful?

a) Hardly at all 18

b) To a small degree 7

c) To a moderate degree 10

d) To a considerable degree 6

e) To a very high degree 15

Question #3 – What should we do with the PiB next year?

a) Make it available next year 32

b) Use it in the laboratory in general 18

c) Discontinue its use 6

Students were also given the opportunity to comment on the PiB system. The following is a summary of the germane comments:

• I think the resource is a good idea. However I am still a fan of having a professor in the lab.

• I used PiB system just about every Friday in the 2nd block. I really found it helpful. I was impressed that it worked so well and that Dr. Moorman was really able to see what we wanted to show him with the camera. It was a really helpful addition to my studying mechanisms because sometimes a group of us would have a discrepancy about what something was/where something was and Dr. Moorman could give us a definitive answer in less than a minute on something that might have never been resolved if we all kept fighting about it together.

• It would be really great it you could have it on the prosected cadaver for a block and then maybe have it on another body (the best body for the internal ab structures, for example...) for another block. I don't know if moving the camera is just too hard, but I think that would be helpful so that at least people wont always just rely on the prosected cadaver.

• I also think that PIB is something that we should try to do with the other universities. I think it would be VERY helpful to be able to speak with profs from other places if that could get set up.

• As far as I'm concerned, the only bad thing about Prof-In-The-Box was that the Internet connection was terrible and it kept cutting out.

• I thought it was very nice of instructors to give up their free time to sit in front of the screen and help us with the cadavers like that. I think it was generally an OK idea. It was better than nothing when you were really unsure about something on the cadaver.

• Very useful and helpful to clarify information.

• Prof-in-the-box was good for checking whether or not your understanding of the location of structures was right.

• I thought the Prof in the Box was a great tool to use. Highly recommended using it again.

• Good for when you have questions.

• It was a good resource

• First of all its fun. Second of all it promotes peer teaching with the reassurance that there is a teacher there just in case there's a real problem.

## Discussion

Four requirements had to be met prior to starting the project and choosing computers for the PiB system: (1) All videoconference transmissions had to be secure (encrypted); (2) The computers had to have built-in, cross-platform videoconference capability so that there was no additional software to buy; (3) cross-platform control of the computer in the laboratory from the office; and (4) The camera in the laboratory had to have sufficient resolution to identify anatomical structures in a video image.

The videoconference transmissions had to be secure to assure the university of compliance with HIPAA (The Health Insurance Portability and Accountability Act of 1996) and to preserve the confidentiality of our donors. This requirement precluded using any of the free commercial instant messaging/videoconference servers such as AIM^®^, MSN^®^, or Yahoo^®^. During the initial development of this project, Apple Computers announced that the next version of OS-X would include an iChat server as an integral part of the OS-X server software. The iChat server is a Jabber^® ^based server and can host both secure and non-secure videoconferences. Jabber^® ^is an open-source cross-platform set of streaming XML protocols and technologies that enable any two computers on the Internet to exchange messages, audio, video, and files in close to real time.

Apple's iChat software is preinstalled on all Apple computers. Microsoft's MSN Messenger is preinstalled on all PCs with the Windows^® ^operating system. However, MSN Messenger can only communicate with the public IM servers and with Microsoft's Live Communications Server software. There are commercial implementations of a Jabber^® ^client for the PC that allow secure videoconferences, but these software packages are not free. The conclusion was that Apple computers had the only built-in software that met the requirements.

In this initial project, it was desirable to have Dr. PiB be as user friendly as possible in the dissection laboratory. This meant the students should not have to be concerned with turning on the system, contacting the faculty member, or switching the cameras to allow the faculty member to see the cadaver. The student should only be concerned with aiming the camera over the cadaver. This meant that Dr. PiB in the dissection laboratory needed to be controlled using the computer in the office. Within the university's intranet, this can be easily accomplished using Apple Remote Desktop (ARD) software. To accomplish this through the university's firewall, the target computer needs a fixed IP address and the appropriate ports need to be enabled on the firewall to allow data throughput to the target computer. With Virtual Network Computing enabled on a Windows based PC, it is also possible to use ARD software to control a PC.

One of the most important aspects of the PiB system is the requirement that the camera in the laboratory have sufficient resolution to identify structures remotely. The iSight camera has 640 × 480 resolution. The iSight camera appeared to have sufficient resolution necessary to identify anatomical structures. However, in order to fill the field of view with an anatomical specimen at a magnification that allows structures to be identified, the working distance of the camera is so short that it precludes the student and camera making simultaneous observations. Although miniDV format cameras have the same resolution as the iSight camera, the way the image is handled results in a higher effective resolution. This means the videoconference image originating from a miniDV camera can be enlarged on the computer screen without loss of resolution making anatomical structures easier to identify. The miniDV camera also had the advantage of a significantly longer working distance, even without using the zoom feature. This meant that the camera could be positioned in a way that did not interfere with the student's ability to see the dissection field while the camera was in place. Interestingly, the students tended to place the camera too close to the cadaver in an attempt to fill the field of view with just the structure they wanted identified. They were routinely asked to move the camera farther from the cadaver to give a larger field of view.

Less than a third of the class used Dr. PiB for help at the prosected cadaver. Several things can explain this low participation rate. Since use of the anatomy dissection laboratory outside of scheduled class hours is not monitored, the number of students that actually use the prosected cadaver is not known. Since Dr. PiB's 'office hours' were on the only afternoon that the students didn't have scheduled classes, the number of students using the prosected cadaver might be a reflection of how many students choose to spend Friday afternoon studying anatomy in the dissection laboratory. 90–95% of the students who came to the prosected cadaver during 'office hours' used Dr. PiB and the students who took advantage of Dr PiB were enthusiastic about the usefulness of the system. Although this might have more to do with students appreciating any extra help provided by the faculty, it suggests that the PiB system represents a viable alternative for providing help in the dissection laboratory when faculty are not able to be physically present. This also supports the idea that a geographically dispersed faculty can provide instruction in the anatomy dissection laboratory using distance-learning technologies. For instance, the University of Medicine and Dentistry of New Jersey has 3 medical schools under its umbrella. If each school had 3 full-time faculty members on-site to teach in the dissection lab, 6 additional faculty members could be available to each school remotely via the PiB system. By judicious scheduling of the 3 gross anatomy courses, all of the lecture and dissection laboratory teaching could be provided by the 9 geographically dispersed faculty employed by one university. Similar collaborations between groups of 3–4 medical schools could provide gross anatomy laboratory experiences for the current numbers of students with 66–75% fewer faculty.

An additional issue that we struggle with in anatomy is getting more clinicians helping in the dissection lab. The presence of clinicians in the laboratory creates a greater sense of relevance for the material being learned. Using the PiB system, clinicians could be available for consultation in the dissection laboratory without leaving the clinics.

The current version of Dr. PiB was fixed in place at one cadaver in the dissection lab. The next version of the system, Dr. PiB version 1.1 will be a mobile unit consisting of the same hardware mounted on a cart equipped with an uninterruptible power supply (battery). Since the computer has an 802.11g wireless card and the entire dissection laboratory has 802.11g wireless coverage, the mobile Dr. PiB can be used anywhere in the lab. This mobile Dr. PiB is being created in response to one of the student's suggestions. The PiB system might have more limitations for helping students at their own cadaver where the quality of the dissection might not be on a par with that of the prosected cadaver. Having a mobile Dr. PiB will allow these limitations to be determined.

The goals of this project were to demonstrate that the cameras and Internet AV software have sufficient resolution and bandwidth to make 'remote' identification of anatomical structures possible and to demonstrate a willingness on the part of the students to receive instruction from faculty at a different geographic location. The results presented here suggest that these goals have been accomplished.

## Conclusion

Many of the functions of a faculty member in the gross anatomy dissection laboratory can be performed 'at a distance' using the PiB system. This suggests that a geographically dispersed faculty could assist in providing instruction in the dissection labs at multiple medical schools without needing to be physically present.

## Competing interests

The author declares that he has no competing interests.

## Pre-publication history

The pre-publication history for this paper can be accessed here:


